# An Atypical Presentation of Cutaneous Angiosarcoma: A Diagnostic Challenge in an End-Stage Renal Disease Patient

**DOI:** 10.7759/cureus.102076

**Published:** 2026-01-22

**Authors:** Sydney L McManus, Ty Theriot, Kayla Rykiel, Allison Pinner, Christopher Haas

**Affiliations:** 1 Dermatology, Louisiana State University Health Sciences Center New Orleans, New Orleans, USA; 2 Medicine/Pediatrics, Louisiana State University Health Sciences Center New Orleans, New Orleans, USA; 3 Medicine, Louisiana State University Health Sciences Center New Orleans, New Orleans, USA

**Keywords:** angiosarcoma, atypical presentation, calciphylaxis, chronic venous stasis, cutaneous angiosarcoma, dermatologic oncology, end-stage renal disease (esrd), multidisciplinary management, soft tissue sarcoma

## Abstract

Angiosarcomas are rare and highly aggressive malignant vascular tumors that are associated with a very poor prognosis. The rarity of the disease, its variable clinical presentations, and differing pathophysiologic mechanisms make diagnosing angiosarcoma a challenge. This case report details a 75-year-old woman with an atypical presentation of cutaneous angiosarcoma in the absence of traditional risk factors. Due to the patient’s history of end-stage renal disease, her initial presentation was highly suspicious for cutaneous calciphylaxis of the left lower extremity. After a thorough workup, this patient likely had malignant transformation of her chronic venous insufficiency. This case underscores the necessity for healthcare providers to consider angiosarcoma in the differential diagnosis of atypical, persistent violaceous nodules and plaques, even in the absence of classic risk factors such as radiation exposure, chronic lymphedema, genetic syndromes, and certain linked carcinogens. A high index of suspicion, combined with timely diagnostic interventions, is vital to address this aggressive malignancy effectively.

## Introduction

Angiosarcomas are rare and highly aggressive malignant vascular tumors that have a heterogeneous clinical profile and account for 1-2% of soft tissue sarcomas [1]. While angiosarcomas are extremely rare, they remain a diagnosis with a poor prognosis, with overall survival between 6 and 16 months after diagnosis [1]. Additionally, the pathophysiology between the different subtypes of angiosarcomas varies widely and is not fully understood. The rarity of the disease, its variable clinical presentations, and differing pathophysiologic mechanisms make diagnosing angiosarcoma a challenge [1,2]. Many times, early cutaneous lesions can be mistaken for benign etiologies, like ecchymoses and hematomas, or infectious etiologies such as cellulitis, resulting in delayed diagnosis [2]. The gold standard for diagnosis requires immunohistochemical evaluation of a tissue sample [2].

Cutaneous angiosarcomas, as seen in this patient, most commonly present as lesions on the head and neck in older men with a median age of 60-71 years old [1]. Risk factors associated with cutaneous angiosarcomas include radiation exposure, chronic lymphedema, genetic syndromes, and other carcinogens (vinyl chloride, arsenic, and thorium dioxide) [1,3]. Women who are diagnosed with angiosarcomas are more likely to have had a history of radiation or lymphedema [3]. Treatments for localized cutaneous angiosarcomas require a multidisciplinary approach, including combinations of surgery with wide negative margins, radiation therapy, chemotherapy, or targeted immunotherapy when appropriate [3]. This case report details an atypical presentation of cutaneous angiosarcoma and highlights the necessity for healthcare providers to consider angiosarcomas in the differential diagnosis of atypical, persistent violaceous nodules and plaques, even in the absence of classic risk factors.

## Case presentation

A 75-year-old woman with a past medical history of end-stage renal disease (ESRD) on hemodialysis three times a week, heart failure with preserved ejection fraction, hyperlipidemia, and chronic venous stasis ulcers presented to the emergency department for a chief complaint of left lower extremity pain, diffuse blistering with associated drainage, and swelling for one week. Over the prior 2-3 months, she noted having developed numerous blisters on the left lower extremity. Initially, these lesions were asymptomatic, but over the previous month, they began to bleed and swell. She endorsed having chronic edema of the lower extremities but denied any trauma to the area or recent infections prior to her symptom onset.

**Figure 1:**
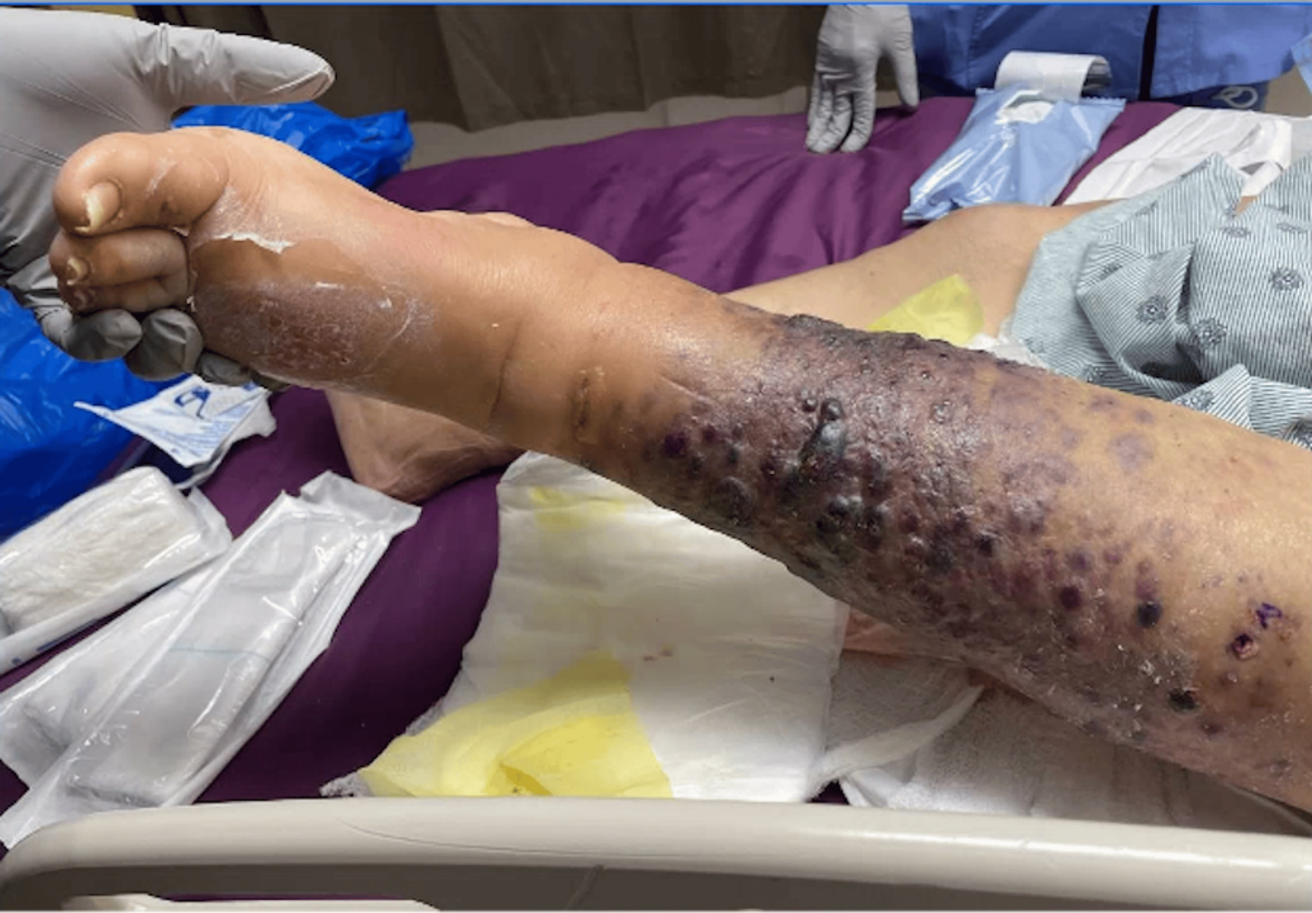
Left lower extremity with multiple violaceous to erythematous tense nodules and plaques with significant left lower extremity edema.

**Figure 2:**
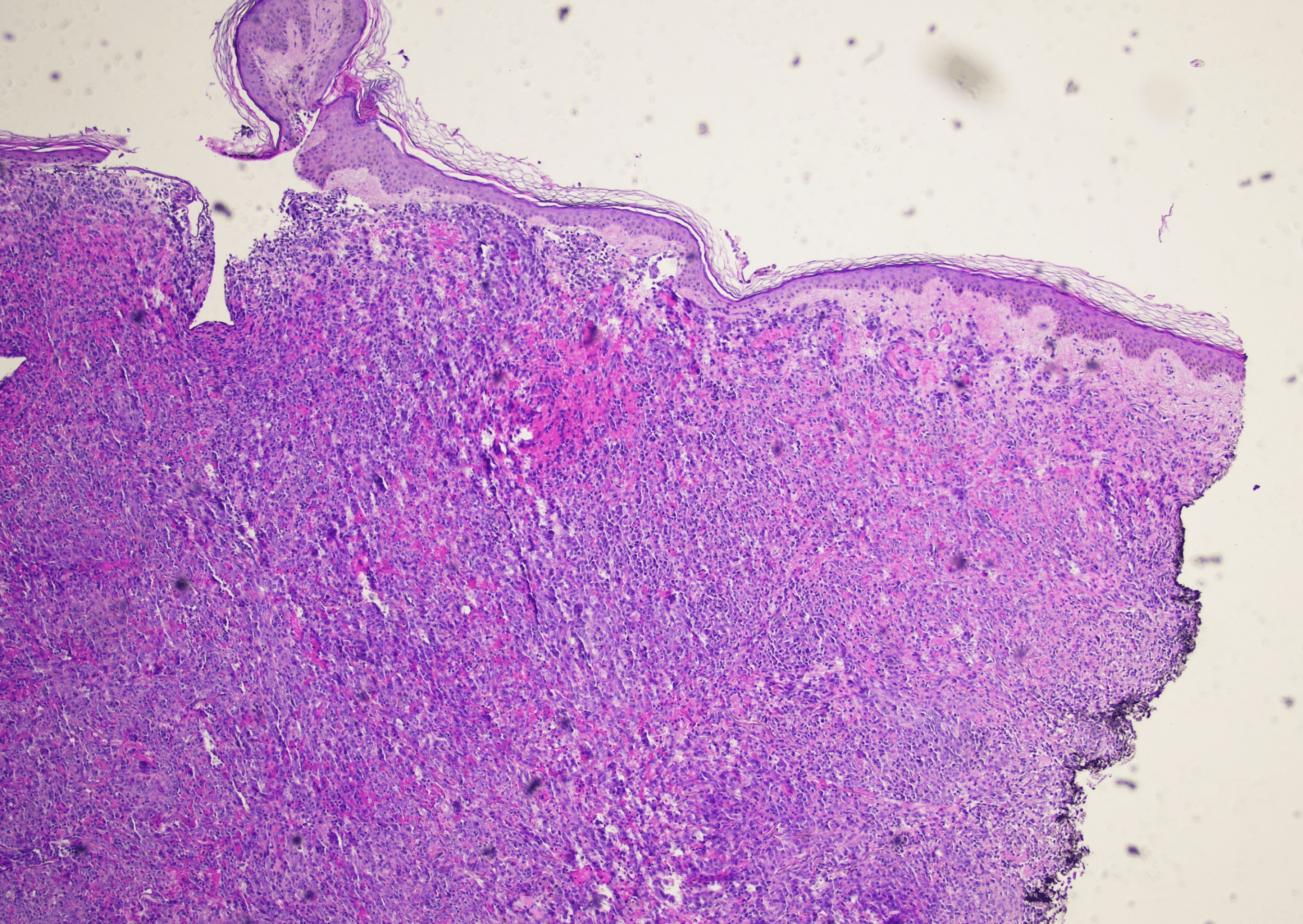
Biopsy of left lower extremity plaque at 4x magnification. Proliferation of atypical cells with bizarre-shaped vascular spaces forming nodules in the dermis.

**Figure 3:**
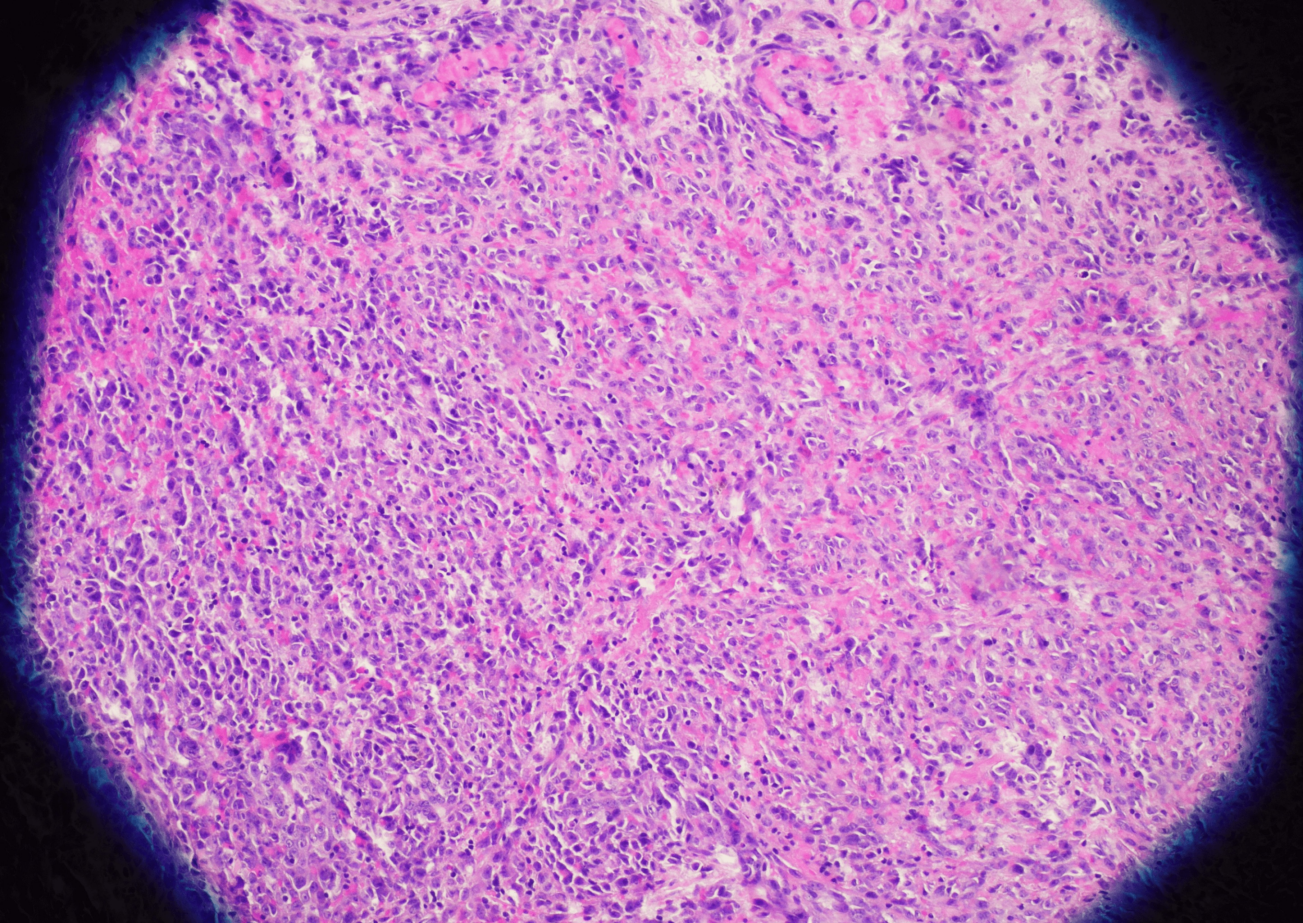
Biopsy of left lower extremity plaque at 10x magnification. Atypical medium-sized pleomorphic cells, hypercellularity, and diffuse infiltrating pattern with red blood cell extravasation present.

**Figure 4:**
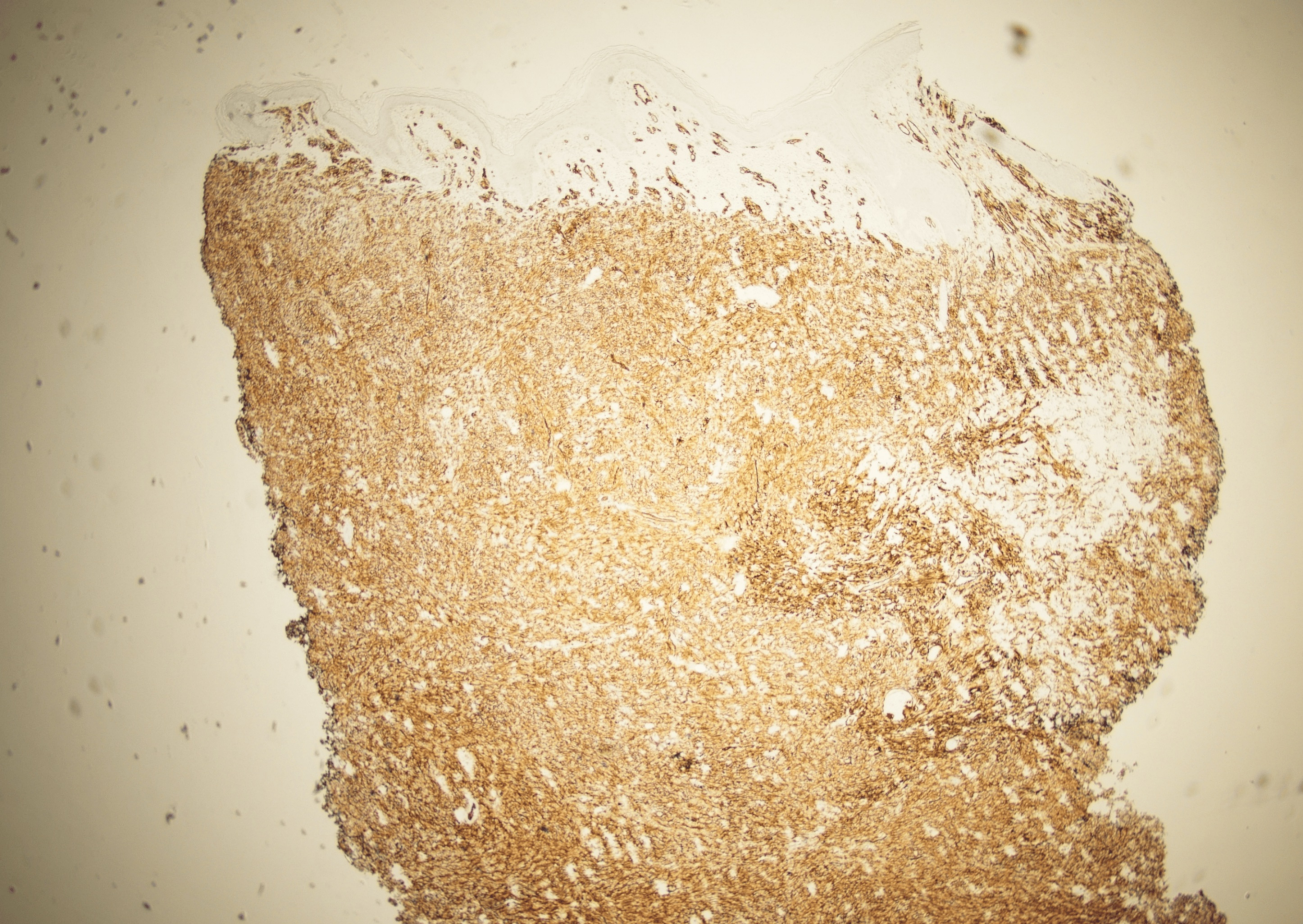
Biopsy of left lower extremity plaque with CD31 staining.

**Figure 5:**
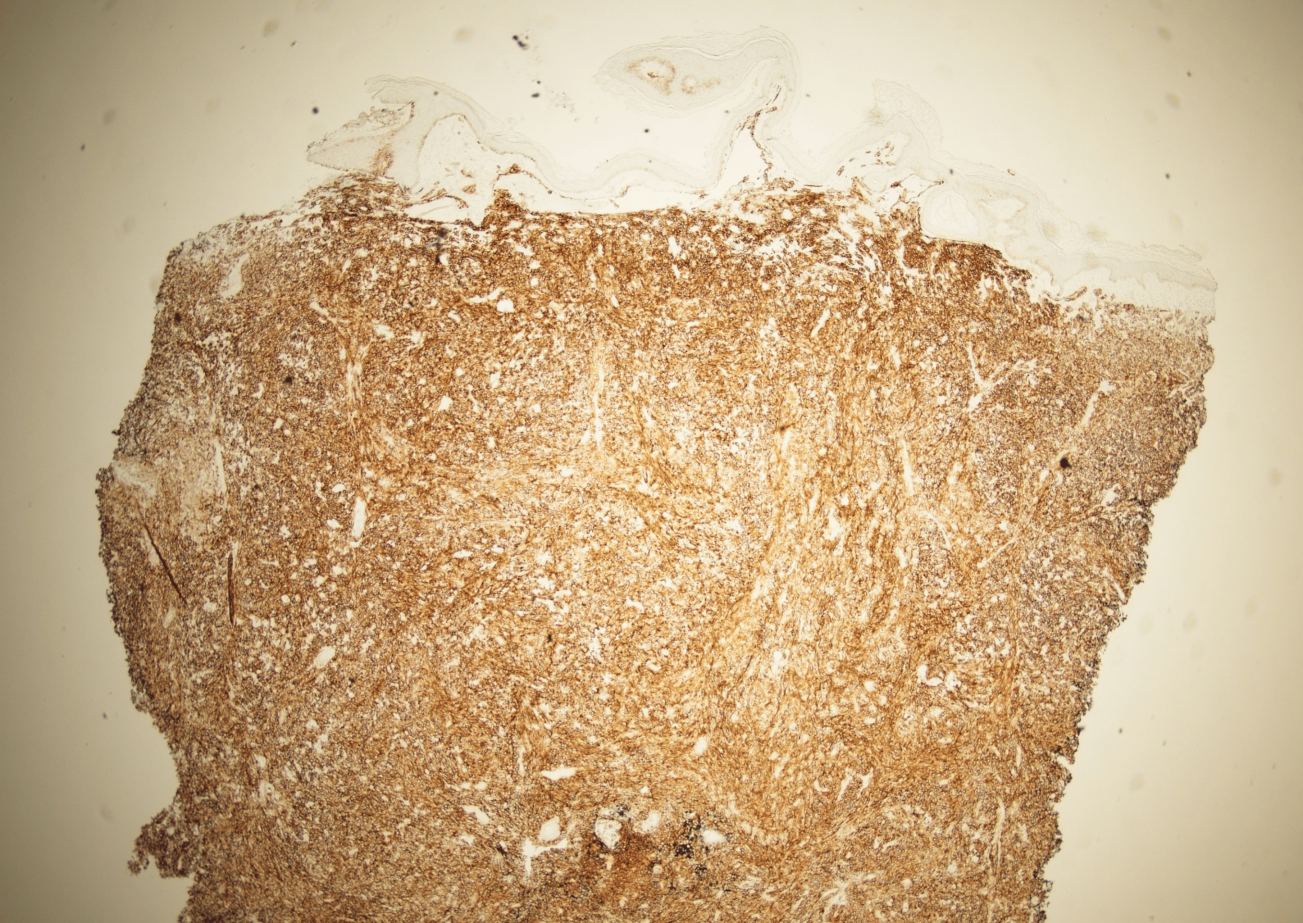
Biopsy of left lower extremity plaque with D240 staining.

Upon presentation, she was afebrile and hemodynamically stable. Physical exam of the left lower extremity revealed multiple violaceous to erythematous tense nodules in addition to nodules that coalesced into plaques with significant left lower extremity edema (Figure 1). Erythrocyte sedimentation rate (ESR) and C-reactive protein (CRP) were obtained to evaluate for potential inflammatory or infectious etiologies. They were elevated to 59mm/hr (reference range: 0-30 mm/hr) and 3.3mg/dL (reference range: <0.9 mg/dL), respectively. While these markers are not diagnostic, they helped lower the threshold for lesion biopsy and further histologic evaluation. CT angiography of the left lower extremity revealed diffuse thickening of the lower extremity without fluid collection. Due to her history of ESRD, cutaneous calciphylaxis was highly suspected at the time of presentation. Further investigating her cutaneous presentation, two telescoping biopsies of her lesions were collected. Histopathology revealed proliferation of atypical cells with bizarre-shaped vascular spaces forming nodules in the dermis (Figure 2). The tumor cells show significant cytologic atypia, hypercellularity, and a diffuse infiltrating pattern into vessels and red blood cell extravasation (Figure 3). The tumor cells stained positive for CD31 and D240 (Figure 4 and Figure 5). AE1/AE3, Melan-A, and S100 stains are unremarkable within the tumor cells, ultimately ruling out squamous cell carcinoma or melanoma. Ki-67 reveals a severely increased mitotic proliferative index in the tumor cells. The pathology was considered too high-grade to consider Kaposi sarcoma as a potential diagnosis. The final pathology report yielded a diagnosis of cutaneous angiosarcoma due to the infiltrative vascular proliferation with marked cellular atypia, red blood cell extravasation, and a high mitotic index. Furthermore, the immunohistochemistry further supports this diagnosis as it demonstrated positivity for vascular markers (CD 31 and D240) and negativity for epithelial and melanocytic markers. The patient was scheduled for follow-up with oncology to discuss options for managing her condition, but she passed away before any definitive management plans were decided.

## Discussion

Given the history of ESRD and clinical presentation of this patient, calciphylaxis was at the top of the differential diagnosis, despite the asymptomatic nature of the lesions. There was a lesser suspicion for angiosarcoma given the distribution to the lower extremities as well as lack of risk factors such as prior radiation, chronic lymphedema, carcinogen exposure, etc. After analysis, histopathological examination revealed significantly atypical endothelial cells with a dense, infiltrating pattern, nodular architecture, and a high mitotic index with a Ki-67 of 90%, which were the most salient features leading to the final diagnosis of cutaneous angiosarcoma. Though no classic risk factors were present, chronic venous stasis and persistent edema could create a localized pro-inflammatory or hypoxic environment conducive to tumorigenesis. Our patient’s laboratory findings, such as elevated inflammatory markers, and imaging findings of lower extremity edema support this hypothesis and parallel similar case reports. While direct evidence linking chronic venous insufficiency to angiosarcomas is limited, clinicians should remain vigilant for malignant transformations in patients with longstanding venous disease [4].

Early stages of angiosarcomas often mimic benign conditions, leading to misdiagnoses and treatment delays. For instance, a 78-year-old male with a persistent scalp lesion was initially treated for a refractory head wound; only after surgical intervention was a diagnosis of scalp angiosarcoma confirmed [5]. Similarly, a 32-year-old woman with a history of bilateral reduction mammoplasty presented with a non-tender breast lump. After repeated aspirations and biopsies, a diagnosis of primary angiosarcoma of the breast was made, highlighting the potential for initial misdiagnosis [6]. It is important that angiosarcoma is considered in patients with atypical presentations, as seen in this patient, due to its highly aggressive nature and low survival rates. Earlier detection and diagnosis may lead to earlier therapeutic initiation and improve patient outcomes.

Managing angiosarcoma in ESRD patients presents unique challenges. While a direct association between ESRD and angiosarcoma is not well-established, there are reports of angiosarcoma arising in vascular access sites of ESRD patients. For example, a kidney transplant recipient developed angiosarcoma at the site of a nonfunctional arteriovenous fistula, suggesting that chronic vascular alterations might contribute to tumorigenesis [7]. Renal impairment may limit the use of certain chemotherapeutic agents due to their nephrotoxic potential. Additionally, surgical interventions must consider the patient's overall health and comorbidities. A multidisciplinary approach is essential to tailor treatment plans that balance efficacy with the patient's renal function and quality of life.

This case emphasizes the necessity for healthcare providers to consider angiosarcoma in the differential diagnosis of atypical, persistent cutaneous lesions, even in the absence of classic risk factors. Early biopsy and histopathological examination are crucial for prompt diagnosis and initiation of appropriate therapy, which may improve patient outcomes.

## Conclusions

In summary, angiosarcoma can present with diverse and atypical manifestations, particularly in patients with comorbid conditions like ESRD and chronic venous insufficiency. Furthermore, clinicians should consider angiosarcoma in the differential diagnosis of atypical, persistent cutaneous lesions regardless of the presence of traditional risk factors. Due to the highly aggressive nature and low survival rates of angiosarcoma, early biopsy and histopathological examination are crucial for prompt diagnosis and initiation of appropriate therapy. Earlier detection and diagnosis may lead to earlier therapeutic initiation and improve patient outcomes. Although this report represents a single case and may limit generalizability, it highlights the importance of astute clinical suspicion and reasoning in similar patient presentations.
